# A family study on first episode of psychosis patients: exploring neuropsychological performance as an endophenotype

**DOI:** 10.1192/j.eurpsy.2022.308

**Published:** 2022-09-01

**Authors:** R. Ayesa-Arriola, N. Murillo-García, A. Díaz-Pons, M. Miguel-Corredera, S. Barrio-Martínez, V. Ortiz-García De La Foz

**Affiliations:** Valdecilla Biomedical Research Institute, Psychiatry, Santander, Spain

**Keywords:** First episode of psychosis, Neurocognitive endophenotype, schizophrénia, First-degree relatives

## Abstract

**Introduction:**

Family studies provide the opportunity to investigate endophenotypes as a powerful neurobiological platform to better understand the underlying neurobiological mechanisms of schizophrenia spectrum disorders. Shared features between the patients and their first-degree relatives may shed some light on the path to identify potential causes of psychosis, and to implement preventive and therapeutic interventions.

**Objectives:**

This study aimed to explore and compare neuropsychological measures in first episodes of psychosis (FEP) patients, their first-degree relatives and healthy controls (HC), participants on the PAFIP-FAMILIES project.

**Methods:**

Statistical analyses were performed using one-way ANOVA, followed by multiple comparisons test where appropriate. Age, sex and years of education were introduced as covariates.

**Results:**

From 387 eligible FEP patients enrolled in a previous cohort, 133 were included. In addition, 244 of their first-degree relatives (146 parents and 98 siblings) and 202 HC participated in this study (see Figure 1). In general, relatives showed an intermediate neuropsychological performance between the HC and the FEP patients (see Figure 2). Specifically, siblings performed similar to HC in the domains verbal memory, visual memory, working memory, motor dexterity and theory of mind, since their values practically overlap those of HC. The parents presented significant deficits, similar to that of the affected individuals, in executive functions and attention domains.

**Figure:**
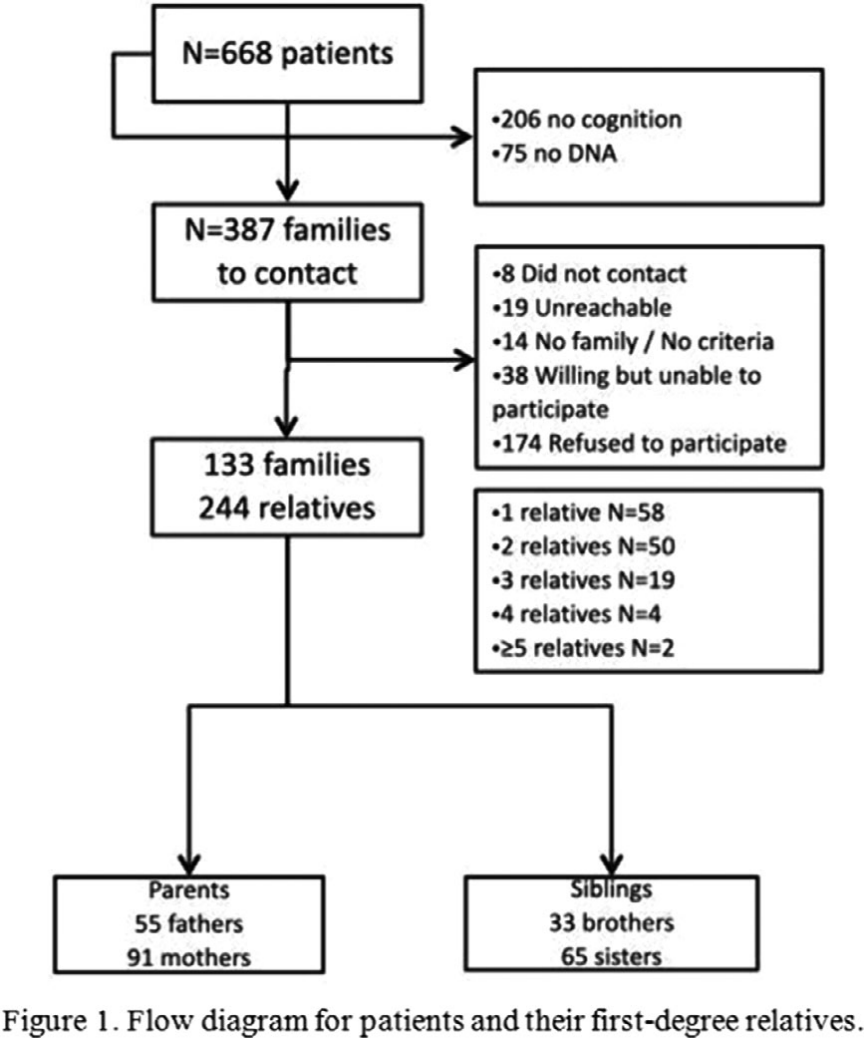

**Figure:**
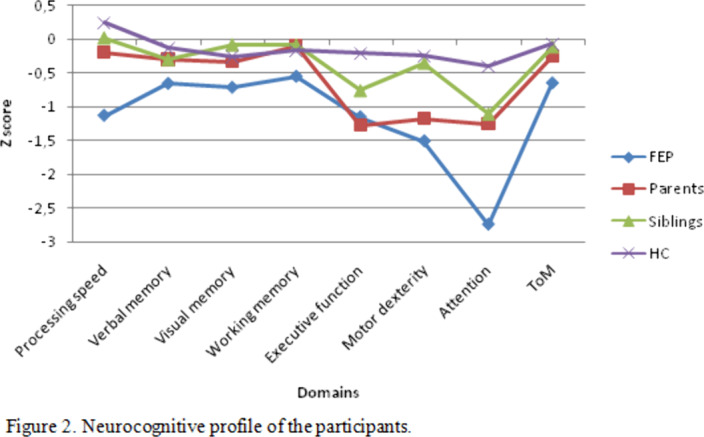

**Conclusions:**

These findings suggest that executive and attention dysfunction might have a greater family aggregation and could be a relevant cognitive endophenotype for psychotic disorders. The study shows the potential of exploring intra-family neuropsychological performance supporting neurobiological and genetic research in schizophrenia.

**Disclosure:**

No significant relationships.

